# A novel AIE-NIR-II nano-contrast agent for monitoring and evaluating kidney transplant

**DOI:** 10.1093/nsr/nwae048

**Published:** 2024-02-05

**Authors:** Yuan-Yuan Zhao, Bokyeong Hwang, Yeju Lee, Juyoung Yoon

**Affiliations:** Department of Chemistry and Nanoscience, Ewha Womans University, South Korea; Department of Chemistry and Nanoscience, Ewha Womans University, South Korea; Department of Chemistry and Nanoscience, Ewha Womans University, South Korea; Department of Chemistry and Nanoscience, Ewha Womans University, South Korea

Kidney transplantation is a life-saving approach for treating end-stage renal diseases (ESRDs) [1]. However, the scarcity of donor kidneys has resulted in an increasing number of ESRD patients being stuck on the transplant waiting list [2]. Improving the survival rate of transplanted kidneys remains a crucial step in addressing the shortage of donor organs. Real-time monitoring of the surgical process with the help of molecular imaging techniques has been well developed in recent years, which has indeed played a crucial role for tumor resection [3–5], while its application in organ transplantation has scarcely been explored. In comparison with traditional tomographic imaging modalities such as computed tomography and magnetic resonance imaging, fluorescence imaging in the second near-infrared (NIR-II) region has emerged as an ideal approach for *in situ* and real-time monitoring of various diagnostic and therapeutic processes [6–8]. The commercial NIR-II contrast agent indocyanine green suffers from poor photostability such that it cannot support the achievement of time-consuming organ transplantation surgery such as kidney transplants. In this regard, NIR-II contrast agents with high brightness, long circulating times and high photostability in aggregates are highly desirable.

**Figure 1.**
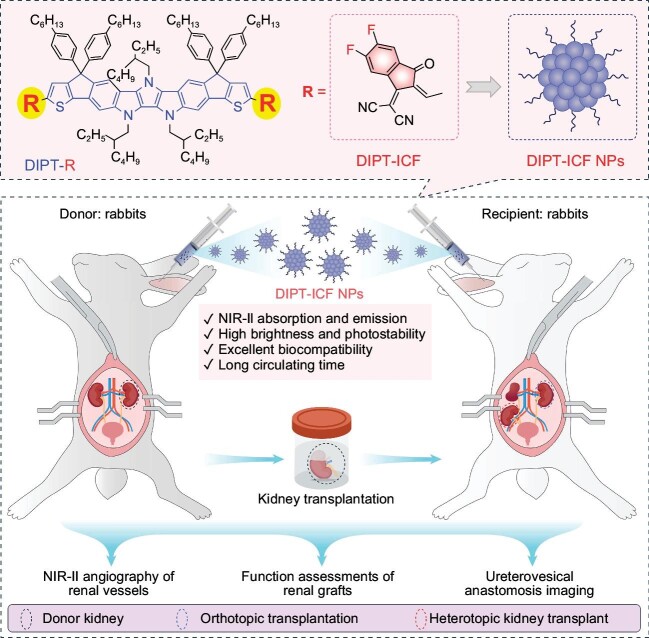
The development of the NIR-II nano-contrast agent (DIPT-ICF NPs) for monitoring the whole process of kidney transplantation. Adapted from Ref. [9].

Researchers from the Chinese University of Hong Kong, Shenzhen (CUHK-Shenzhen) recently developed a novel NIR-II aggregation-induced emission (AIE) contrast agent (DIPT-ICF) and successfully explored its application in monitoring and evaluating the whole

process of kidney transplantation (Fig. 1) [9]. Being different from traditional AIE luminogens (AIEgens) with twisted molecular structures, DIPT-ICF displays a coplanar structure with an ultra-high molar absorptivity of 1.79 ± 0.03 × 10^5^ M^−1^ cm^−1^ at 820 nm, which is 1.2 times higher than that of the commercial IR-26. Moreover, the absolute photoluminescence quantum yield (PLQY) of the DIPT-ICF in tetrahydrofuran solution was determined to be 0.09%, resulting in a calculated brightness of 161.1 M^−1^ cm^−1^. It is ∼2.2 times brighter than IR-26 in the single-molecule state. The coplanar AIEgen DIPT-ICF demonstrates a new approach to developing a superior light-harvesting AIEgen that is highly suitable for the fabrication of high-brightness nano-contrast agents. The luminescence of DIPT-ICF nanoparticles (NPs) is also outperforming other commercial fluorescence contrast agents, e.g. IR1061 NPs. Furthermore, its prolonged circulating time and elevated photostability have extended the optimal imaging window period, reducing the intraoperative frequency of contrast agent administration.

With this promising AIE nano-contrast agent, the research team conducted comprehensive monitoring and assessment of various stages in model animal kidney transplant surgeries. In the surgery of living-donor nephrectomy, NIR-II angiography of renal vasculature using DIPT-ICF NPs helps surgeons to visualize the renal vascular branches and their anatomical locations, thus avoiding iatrogenic donor kidney injury. Moreover, real-time observation of surgical complications in vascular and ureterovesical anastomosis is achieved through NIR-II angiography and ureterography with the help of the DIPT-ICF NPs contrast agent. More significantly, by leveraging the novel AIE nano-contrast agent, researchers have introduced a groundbreaking method for non-invasive diagnosis of glomerular filtration barrier injuries through real-time urine fluorescence detection. This approach holds promise for identifying and assessing the severity of renal damage associated with kidney transplant rejection reactions.

In summary, this study presents a crucial and viable strategy for monitoring and evaluating renal transplantation in the clinic.
